# Extracting seizure frequency from epilepsy clinic notes: a machine reading approach to natural language processing

**DOI:** 10.1093/jamia/ocac018

**Published:** 2022-02-22

**Authors:** Kevin Xie, Ryan S Gallagher, Erin C Conrad, Chadric O Garrick, Steven N Baldassano, John M Bernabei, Peter D Galer, Nina J Ghosn, Adam S Greenblatt, Tara Jennings, Alana Kornspun, Catherine V Kulick-Soper, Jal M Panchal, Akash R Pattnaik, Brittany H Scheid, Danmeng Wei, Micah Weitzman, Ramya Muthukrishnan, Joongwon Kim, Brian Litt, Colin A Ellis, Dan Roth

**Affiliations:** Department of Bioengineering, School of Engineering and Applied Sciences, University of Pennsylvania, Philadelphia, Pennsylvania, USA; Center for Neuroengineering and Therapeutics, University of Pennsylvania, Philadelphia, Pennsylvania, USA; Center for Neuroengineering and Therapeutics, University of Pennsylvania, Philadelphia, Pennsylvania, USA; Department of Neurology, Penn Epilepsy Center, Perelman School of Medicine, University of Pennsylvania, Philadelphia, Pennsylvania, USA; Department of Neurology, Penn Epilepsy Center, Perelman School of Medicine, University of Pennsylvania, Philadelphia, Pennsylvania, USA; Center for Neuroengineering and Therapeutics, University of Pennsylvania, Philadelphia, Pennsylvania, USA; Department of Bioengineering, School of Engineering and Applied Sciences, University of Pennsylvania, Philadelphia, Pennsylvania, USA; Center for Neuroengineering and Therapeutics, University of Pennsylvania, Philadelphia, Pennsylvania, USA; Department of Bioengineering, School of Engineering and Applied Sciences, University of Pennsylvania, Philadelphia, Pennsylvania, USA; Center for Neuroengineering and Therapeutics, University of Pennsylvania, Philadelphia, Pennsylvania, USA; Department of Bioengineering, School of Engineering and Applied Sciences, University of Pennsylvania, Philadelphia, Pennsylvania, USA; Center for Neuroengineering and Therapeutics, University of Pennsylvania, Philadelphia, Pennsylvania, USA; Department of Biomedical and Health Informatics, Children’s Hospital of Philadelphia, Philadelphia, Pennsylvania, USA; Department of Bioengineering, School of Engineering and Applied Sciences, University of Pennsylvania, Philadelphia, Pennsylvania, USA; Center for Neuroengineering and Therapeutics, University of Pennsylvania, Philadelphia, Pennsylvania, USA; Department of Neurology, Penn Epilepsy Center, Perelman School of Medicine, University of Pennsylvania, Philadelphia, Pennsylvania, USA; Department of Neurology, Penn Epilepsy Center, Perelman School of Medicine, University of Pennsylvania, Philadelphia, Pennsylvania, USA; Department of Neurology, Penn Epilepsy Center, Perelman School of Medicine, University of Pennsylvania, Philadelphia, Pennsylvania, USA; Department of Neurology, Penn Epilepsy Center, Perelman School of Medicine, University of Pennsylvania, Philadelphia, Pennsylvania, USA; Department of Bioengineering, School of Engineering and Applied Sciences, University of Pennsylvania, Philadelphia, Pennsylvania, USA; Center for Neuroengineering and Therapeutics, University of Pennsylvania, Philadelphia, Pennsylvania, USA; The General Robotics, Automation, Sensing and Perception Laboratory, School of Engineering and Applied Sciences, University of Pennsylvania, Philadelphia, Pennsylvania, USA; Department of Bioengineering, School of Engineering and Applied Sciences, University of Pennsylvania, Philadelphia, Pennsylvania, USA; Center for Neuroengineering and Therapeutics, University of Pennsylvania, Philadelphia, Pennsylvania, USA; Department of Bioengineering, School of Engineering and Applied Sciences, University of Pennsylvania, Philadelphia, Pennsylvania, USA; Center for Neuroengineering and Therapeutics, University of Pennsylvania, Philadelphia, Pennsylvania, USA; Department of Neurology, Penn Epilepsy Center, Perelman School of Medicine, University of Pennsylvania, Philadelphia, Pennsylvania, USA; Department of Electrical and Systems Engineering, School of Engineering and Applied Sciences, University of Pennsylvania, Philadelphia, Pennsylvania, USA; Department of Computer and Information Science, School of Engineering and Applied Sciences, University of Pennsylvania, Philadelphia, Pennsylvania, USA; Department of Computer and Information Science, School of Engineering and Applied Sciences, University of Pennsylvania, Philadelphia, Pennsylvania, USA; Department of Bioengineering, School of Engineering and Applied Sciences, University of Pennsylvania, Philadelphia, Pennsylvania, USA; Center for Neuroengineering and Therapeutics, University of Pennsylvania, Philadelphia, Pennsylvania, USA; Department of Neurology, Penn Epilepsy Center, Perelman School of Medicine, University of Pennsylvania, Philadelphia, Pennsylvania, USA; Center for Neuroengineering and Therapeutics, University of Pennsylvania, Philadelphia, Pennsylvania, USA; Department of Neurology, Penn Epilepsy Center, Perelman School of Medicine, University of Pennsylvania, Philadelphia, Pennsylvania, USA; Department of Computer and Information Science, School of Engineering and Applied Sciences, University of Pennsylvania, Philadelphia, Pennsylvania, USA

**Keywords:** electronic medical record, natural language processing, epilepsy, question-answering

## Abstract

**Objective:**

Seizure frequency and seizure freedom are among the most important outcome measures for patients with epilepsy. In this study, we aimed to automatically extract this clinical information from unstructured text in clinical notes. If successful, this could improve clinical decision-making in epilepsy patients and allow for rapid, large-scale retrospective research.

**Materials and Methods:**

We developed a finetuning pipeline for pretrained neural models to classify patients as being seizure-free and to extract text containing their seizure frequency and date of last seizure from clinical notes. We annotated 1000 notes for use as training and testing data and determined how well 3 pretrained neural models, BERT, RoBERTa, and Bio_ClinicalBERT, could identify and extract the desired information after finetuning.

**Results:**

The finetuned models (BERT_FT_, Bio_ClinicalBERT_FT_, and RoBERTa_FT_) achieved near-human performance when classifying patients as seizure free, with BERT_FT_ and Bio_ClinicalBERT_FT_ achieving accuracy scores over 80%. All 3 models also achieved human performance when extracting seizure frequency and date of last seizure, with overall F_1_ scores over 0.80. The best combination of models was Bio_ClinicalBERT_FT_ for classification, and RoBERTa_FT_ for text extraction. Most of the gains in performance due to finetuning required roughly 70 annotated notes.

**Discussion and Conclusion:**

Our novel machine reading approach to extracting important clinical outcomes performed at or near human performance on several tasks. This approach opens new possibilities to support clinical practice and conduct large-scale retrospective clinical research. Future studies can use our finetuning pipeline with minimal training annotations to answer new clinical questions.

## INTRODUCTION

The electronic health record (EHR) is an ever-growing resource of documents created by healthcare providers to monitor the wellbeing of patients. Among such documents are progress notes, which are unstructured or minimally structured free-text documents that clinicians use to record interactions with patients. Progress notes contain the patient’s medication list, general wellbeing, diagnoses, treatments, and disease progression.^1^ The EHR is a source of information and data for large-scale retrospective clinical research^2–4^; instead of following cohorts prospectively for extended periods of time, researchers can look backwards through the EHR using information gathered during patient visits to answer clinical questions. Such retrospective studies may also be cheaper to conduct than the current costly prospective standard of research,^2^ which often costs thousands of dollars per subject.^5–8^

However, manually extracting information from the EHR can be time consuming and impractical. Automated text mining methods from natural language processing (NLP), a field of computer science dedicated toward teaching machines to read and comprehend human language, have attempted to mitigate this problem. Recent strides in NLP have created pretrained language models, based largely on the Transformer architecture, with unprecedented abilities to understand unstructured text and generalize across a wide range of tasks and domains.^9–11^ Transfer learning is a key advantage to these models, which are initially pretrained on a large text corpus to obtain a general understanding of language. They can then be finetuned on small datasets to be adapted toward specific tasks or domains. Already, such models have shown strong flexibility and accuracy in specialized domains. For example, the Bio_ClinicalBERT model demonstrated high accuracy and F_1_ scores for various clinical NLP benchmarking tasks.^12^

In the clinical care of patients with epilepsy, among the most important markers of clinical severity and response to treatment are (1) the presence or absence of seizures (seizure freedom) and (2) the frequency of seizures over a given time, generally within the past year or since the last office visit. These outcome measures are challenging for automated extraction from text. For example, paroxysmal events at irregular intervals with dynamic temporal components preclude simple static phenotypic classification of patients. In this study, we developed a novel NLP pipeline to automatically extract seizure freedom and seizure frequency from clinical notes at the University of Pennsylvania Health System (UPHS), validated against human annotations. Our novel pipeline uses a combination of existing pretrained models, publicly available finetuning tools, and a relatively small amount of task-specific annotation; we expect that it will be generalizable to a wide range of research questions that require extracting and classifying text from clinical notes.

## MATERIALS AND METHODS

### Document curation

This retrospective study was approved by the Institutional Review Board of the University of Pennsylvania with a waiver of informed consent. We obtained nearly 79 000 progress notes for patients with epilepsy who visited UPHS between January 1, 2015 and December 31, 2018. We filtered for notes authored by 8 epilepsy specialists, and extracted text from these notes as “paragraphs” to improve the probability that the text involved epileptic events; we truncated these paragraphs to better fit within the maximum sequence length of the models (512 tokens) and appended the date each note was written to the front of each paragraph (see Supplementary Methods). We were interested in identifying patient seizure freedom and extracting their seizure frequencies. We formulated this problem as a question-answering task, where a model attempts to answer a series of questions given a context. From these paragraphs, we asked 3 questions (Table 1): (Q1) “has the patient had recent seizures,” (Q2) “how often does the patient have seizures,” and (Q3) “when was the patient’s most recent seizure.” For question 1, we defined “recent seizures” as occurring either (1) since the last office visit, or (2) in the 1 year before the note was written, whichever was more recent. Note that these questions require distinct paradigms: the first classifies patients as being seizure-free using all available information in the note, while the latter 2 extract the span of text in the paragraph that best answers them. Additionally, an answer to any or all these questions may not exist in a given paragraph.

**Table 1.** Questions and corresponding task paradigm for the question-answering task

Question	Task paradigm
Q1: “Has the patient had recent seizures?”	Classification
Q2: “How often does the patient have seizures?”	Text extraction
Q3: “When was the patient’s most recent seizure?”	Text extraction

### Annotation

We recruited 15 annotators to collectively mark 1000 paragraphs in a manner that answered the 3 questions. This number of documents was chosen to balance annotation time with having sufficient dataset size for model training and evaluation. Documents were chosen randomly but with a uniform distribution across the 8 note authors, such that each author wrote 125 paragraphs, to evenly capture their representative styles. Annotations were performed on the INCEpTION platform.^13^ Our 15 annotators consisted of 7 clinicians (epilepsy nurse practitioners, neurology residents, epilepsy fellows, and attending physicians), and 8 nonclinicians (graduate students or personnel working in a lab focused on epilepsy-related research). We created 5 annotation groups, each with 200 unique paragraphs. We divided our annotators between these groups such that each group had at least 1 clinician and at least 1 nonclinician annotator, and that each document would have 3 independent sets of annotations.

Once all annotations were completed, annotations were merged and adjudicated. Disagreements between annotations for each paragraph were settled in most cases by majority voting between the annotations. On occasions where no majority could be reached, 2 of the authors (KX and CAE) together manually adjudicated the annotation. The 5 groups of 200 adjudicated paragraphs were merged into a single dataset of 1000 paragraphs defined as the “ground truth” annotations.

We calculated interrater reliability among the human annotators using Cohen’s Kappa (κ) for the classification question,^14^ and F_1_ score for the text extraction questions. κ measures the observed agreement between annotators, offset by their expected agreement due to chance. By convention, a κ above 0.81 indicates “near perfect” agreement.^15^ The F_1_ score measures the amount of text overlap; an F_1_ score of 1.0 indicates the spans of text are identical, whereas an F_1_ of 0 indicates no intersection. We calculated the F_1_ score in 2 ways: a paired F_1_, which shows annotator consistency when 2 annotators agree on some amount of text, and an overall F_1_, which includes all annotations for a particular question in each note.

### Model finetuning and evaluation

We randomly split the annotated paragraphs into a training set of 700 paragraphs, and a testing set of 300 paragraphs. We targeted 3 models: the original BERT,^9^ Bio_ClinicalBERT,^12^ which has additional pretraining on clinical and biomedical texts, and RoBERTa,^16^ a version of BERT with improved pretraining objectives, including dynamic masking without next-sentence-prediction over significantly more data, and better hyperparameters, such as a larger batch size and a larger tokenization vocabulary. We selected BERT to serve as a useful baseline, and Bio_ClinicalBERT and RoBERTa due to its improved performance on clinical benchmarking tasks, and its consistently high performance across several general NLP tasks, respectively. Because the classification question (Q1) and text extraction questions (Q2 and Q3) constituted distinct task paradigms, they required 2 separate models, 1 for each type of task.

**Figure 1.**
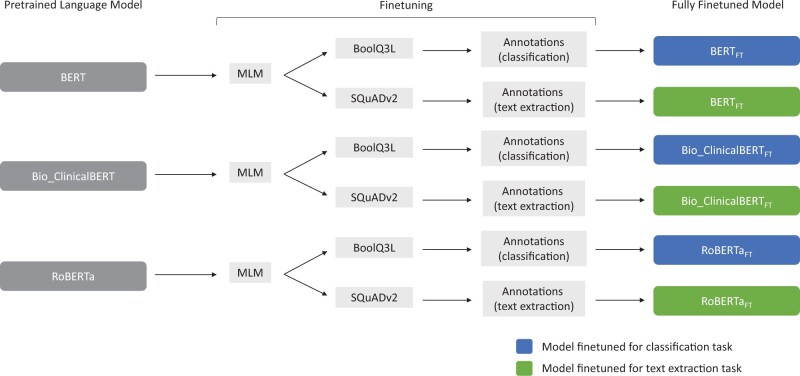
Schematic methods detailing the annotation and finetuning pipeline. Pretrained language models are finetuned using masked language modeling by exposing them to 78 000 unannotated clinical notes; task specific datasets (SQuADv2 and BoolQ3L); and the training set of annotated notes. This process was repeated for 5 different seeds.

Our fine-tuning pipeline (Figure 1) followed the insights of Han and Eisenstein^17^ and Soni and Roberts^18^: using a single pretrained model, we first performed unsupervised domain adaptation with masked language modeling (MLM) using the 78 000 progress notes that were not selected for annotation. We then performed task-specific finetuning with the resultant model in 2 separate instances on publicly available English datasets—1 on BoolQ3L for the classification task,^19^ and the other on SQuADv2 for the text extraction task,^20^ resulting in a pair of models. We concluded model training with supervised finetuning on the training set of the human-annotated medical notes. The order of our pipeline’s steps followed and combined the order of finetuning steps used in the 2 aforementioned studies.^17^^,^^18^ We repeated this pipeline 5 times for each of the 3 starting models with a set of arbitrarily chosen seeds to allow for estimates of the variability of each model’s performance. We denote the models finetuned using the full pipeline as BERT_FT_, Bio_ClinicalBERT_FT_, and RoBERTa_FT_, for the models finetuned from BERT, Bio_ClinicalBERT, and RoBERTa, respectively. We performed ablation studies by removing each step of our pipeline (1 at a time while leaving the other steps unchanged) to evaluate their individual impacts on model performance; as before, we used 5 arbitrarily chosen seeds for each ablation experiment.

We evaluated the performance relative to the ground truth annotations using the accuracy metric for the classification question (defined as correct classifications divided by total classifications), and the F_1_ text overlap score for the text extraction questions. As before, the F_1_ score is based purely on the overlap between spans of text. We set the F_1_ and accuracy scores so that they lie on the same 0–1 scale. We used Huggingface Transformers,^21^ a Python library for neural NLP models, to perform finetuning and used their evaluation methods or the official SQuADv2 evaluation script when appropriate to determine the model’s performance.^20^ For all finetuning steps, we used Huggingface’s default and supplied hyperparameters and did not perform any hyperparameter optimization. We set the maximum sequence length of the 3 models to 512 tokens to capture as much information at once as possible. We assessed human annotator correctness by comparing each individual’s annotations against the final ground truth annotations. Because the NLP models made 1 prediction per question per note, we selected the annotation closest in proximity to the ground truth annotation to make a direct comparison in cases where a human annotator made more than 1 annotation for a given question. If multiple ground truths existed for a given question, the annotation with the highest similarity to any of the ground truths was selected.

## RESULTS

### Interrater reliability and ground truth annotations

**Figure 2.**
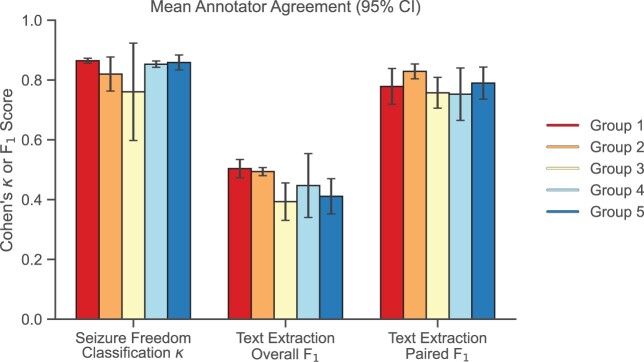
Mean intragroup Cohen’s κ and F_1_ scores with 95% confidence intervals. Annotators within each group were compared pairwise and demonstrated high agreement across all groups. κ was calculated using annotator classification values (Q1). F_1_ was calculated using annotator text extractions (Q2 and Q3).

For the classification question, agreement among human annotators was consistently in the “near perfect” range (average pairwise κ = 0.82, with 13/17 [76%] pairs having κ > 0.8). For the text extraction questions, the paired F_1_ had an average pairwise value of 0.79 when annotators identified overlapping spans of text. The overall F_1_, which considered all annotations per note, had an average pairwise value 0.44 (Figure 2). We found that there was no difference in human annotator correctness between clinician and nonclinician annotators.

The final dataset consisted of ground truth annotations for 1000 notes. For the classification question (Q1), 61.8% of notes were classified as having recent seizures, 30.3% as not having recent seizures, and 7.8% had no answer. For the text extraction questions, 35.5% of notes contained a statement of seizure frequency (Q2) and 49.5% of notes contained a statement of most recent seizure occurrence (Q3).

### Evaluating model performance

**Figure 3.**
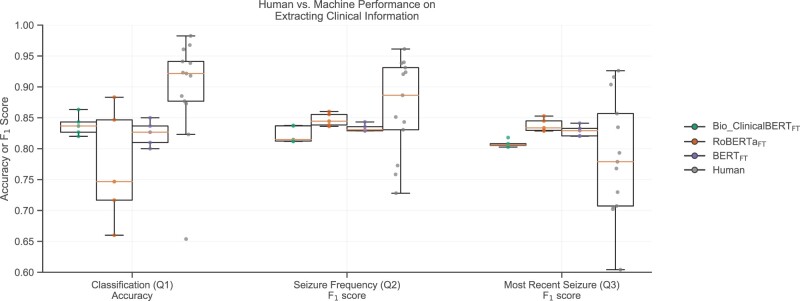
A comparison between the accuracy and F_1_ score of human and machine predictions. Machines achieved near human performance on the classification question, and human-like performance on the text extraction questions. Gray points denote individual annotator performance, whereas green, orange, and purple points denote individual Bio_ClinicalBERT_FT_, RoBERTa_FT_, and BERT_FT_ seeds, respectively. Box plots show median and quartile ranges of values.

Figure 3 shows the performance of each of the 3 fine-tuned models as well as human performance on each of our question-answering tasks, compared with ground truth annotations. For the classification question (Q1), Bio_ClinicalBERT_FT_ and BERT_FT_ each showed strong and robust performances across 5 trials, with Bio_ClinicalBERT_FT_ achieving the highest median (0.837) accuracy, with a maximum of 0.863. RoBERTa_FT_ showed more variability across trials and the lowest median accuracy (0.747). We performed 2-sided Mann-Whitney *U* tests between human annotators and the models with the null hypothesis that the model and human accuracies came from the same distribution. We rejected this hypothesis for the models (*P* values .019, .019, and .014 for Bio_ClinicalBERT_FT_, RoBERTa_FT_, and BERT_FT_, respectively), indicating that they were slightly worse than humans at classifying patients (difference in median accuracy relative to that of humans of −0.085, −0.175, −0.095 for Bio_ClinicalBERT_FT_, RoBERTa_FT_, and BERT_FT_, respectively) despite the excellent overall performance of BERT_FT_ and Bio_ClinicalBERT_FT_.

For the 2 text extraction questions (Q2 and Q3), our models showed stable performance across 5 trials, with RoBERTa_FT_ achieving the highest median (0.845 and 0.834) and maximum F_1_ values (0.860 and 0.853). Again, we performed 2-sided Mann-Whitney *U* tests between the F_1_ scores of our models and our human annotators. These tests failed to reject the null hypothesis for either question, indicating no significant difference in performance in comparison to human annotators (Figure 3).

### Factors that contribute toward model performance

**Figure 4.**
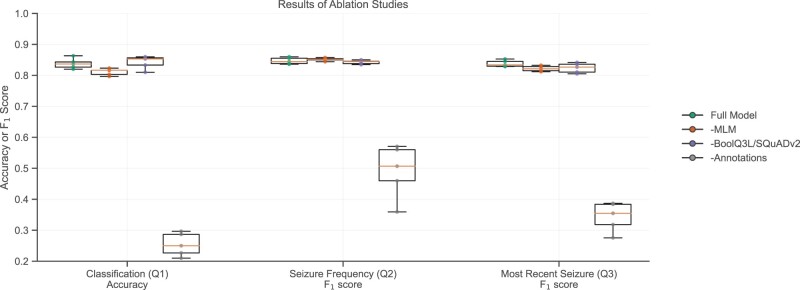
Contributions of the individual steps in the fine-tuning pipeline toward model performance. The masked language modeling step and Annotations influenced final model performance. Bio_ClinicalBERT and RoBERTa were finetuned for classification and text extraction, respectively, with the same seeds. A specified step was removed from the finetuning pipeline in each experiment. Box plots show median and quartile ranges of values.

We conducted ablation studies to investigate the individual impacts of each step of the finetuning pipeline. We used Bio_ClinicalBERT and RoBERTa as the starting models for the classification question (Q1) and text extraction questions (Q2 and Q3), respectively, as they had the best performance on the respective tasks. We first removed the MLM step (-MLM), keeping the task-specific and annotated training datasets, then removed the task-specific datasets (-SQuADv2, -BoolQ3L), keeping the MLM task and the annotated training dataset, and finally removed the annotated training dataset (-Annotations), keeping the MLM task and the task-specific datasets. We performed 2-sided Mann-Whitney *U* tests comparing the ablation experiments to the full finetuning pipeline with the null hypothesis that the ablated models and the full model have equivalent performance. For the classification question, we rejected the null hypothesis for the models finetuned without MLM or annotations (*P* values = .021 and .008, respectively), indicating that those 2 factors were significant contributors to the models’ performance. For the seizure frequency question, we found that only the annotations had a significant contribution to the models’ performance (*P* value = .0079). For the most recent occurrence question, both MLM and annotations were significant contributors to model performance (*P* values = .032 and .008, respectively; Figure 4). Overall, it is evident that the annotated training dataset was the most significant step in the training pipeline, and that the MLM step was occasionally helpful.

**Figure 5.**
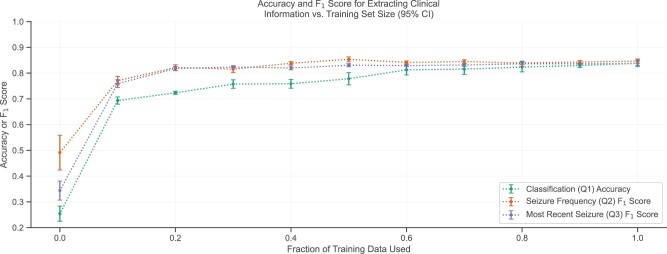
Influence of training set size on mean model performance with 95% confidence intervals. Most of the models’ improvements occurred within the first 10% of the training data. Bio_ClinicalBERT and RoBERTa were finetuned for classification and text extraction, respectively, with the same seeds. Various annotated training set sizes were used for the final finetuning step.

We next asked how much annotated training data were ultimately required. We split the annotated training dataset into cumulative tenths and applied the fine-tuning pipeline with 5 seeds using these subdatasets on Bio_ClinicalBERT and RoBERTa for classification and text extraction, respectively. Using the first 10% of the training dataset (ie, 70 annotated notes), we achieved 82.7% of the final accuracy for the classification question, and 91.0% and 90.6% of the final F_1_ scores for the 2 text extraction questions (Figure 5).

We repeated the above analyses after stratifying notes according to whether an answer existed to the text extraction questions (Q2 and Q3). We found that all language models were very accurate at identifying notes where no answer existed. In notes where an answer existed, performance was lower, although this was true for both the NLP models and the human annotators, with the models performing better than most human annotators (see Supplementary Results).

Finally, we manually reviewed instances where our model failed to predict the correct classification or failed to extract the correct span of text and we identified patterns among the errors. We analyzed the predictions of Bio_ClinicalBERT_FT_ for classification, and RoBERTa_FT_ for text extraction, both with the same best overall seed. We found that the leading cause of errors was due to too much irrelevant information, such as outdated seizure-related information and/or information unrelated to epilepsy in the paragraph, which resulted in the model becoming “confused,” and selecting, for example, an outdated seizure frequency. Additionally, we found that the models had trouble performing higher-order logic, including temporal reasoning. For example, the model had trouble classifying paragraphs that mentioned the patient having an event on a specific date, but being seizure-free from a more recent date, for example, “The patient had a seizure cluster on 4/13. They have been seizure free since May.” The model also failed to perform implicit semantic reasoning; for example, the statement “the patient has been seizure-free since June” was interpreted by human annotators to mean that the patient’s most recent seizure was in June. However, the model consistently failed to identify such statements as indicating a patient’s last event (Q3). Overall, it is evident that many of the prediction errors occurred in an understandable and nonrandom fashion (Table 2).

**Table 2.** Reasons for erroneous predictions

Reason for consistent errors	Number of errors			
	Total	Classifying seizure freedom (Q1)	Extracting seizure frequency (Q2)	Extracting date of last seizure (Q3)
Too much historical or irrelevant information *For example, “In 2002 they began having convulsions, with frequency 3/month.”* *For example, “Patient with history of coronary artery disease… stent thrombosis, prostate cancer… readmitted after a fall… worsening subdural hematoma and rightward subfalcine herniation…”*	44	18	8	18
Many relevant events with different information *For example, “No seizures with loss of awareness since last visit. Continued probable simple partial seizures frequently.”*	8	8	0	0
Identified irrelevant phenomena or failed to identify relevant phenomena from symptoms *For example, “At least daily stomach spasms”*	10	1	7	2
Unusual time anchor *For example, “Occur on the first day of their menses.”*	3	0	3	0
Temporal reasoning *For example, “3 months ago, they had three seizures in July. No seizure since that time.”* *For example, “Not had significant myoclonus since their early 30s.”*	11	9	0	2
Semantic reasoning *For example, “… seizure free for the past 3 months.”*	6	0	1	5

## DISCUSSION

In this work, we developed a finetuning pipeline for NLP language models to improve their understanding of clinical notes for patients with seizures. We demonstrated that models finetuned using these methods can accurately extract seizure-related information from epilepsy notes to a high degree of correctness, rivaling that of a human. We identified consistent patterns of errors in our models and determined how each step of our finetuning pipeline helped to improve the models’ performance. Finally, we note that our methods can be used to answer different questions with minimal amounts of new data.

Our findings demonstrate the ability of machine systems to understand medical text practically and accurately. Our models can be used to support clinical decision-making by rapidly extracting important markers of epilepsy severity and treatment response from unstructured patient histories. Additionally, our generalizable pipeline can be used to conduct retrospective studies, at scale, across a wide range of clinical research questions in a fraction of the time and at a fraction of the cost required for prospective studies.

Among human annotators, we found excellent agreement for the classification question, but poorer agreement overall for the text extraction questions. However, because we triple-annotated all notes and used majority adjudication in most cases, the paired F_1_ score gives a meaningful estimate of the reliability of annotations given that 2 annotators selected similar text spans. The lower F_1_ values when accounting for all annotations indicates that double or triple-annotating notes is still preferable because individual annotations in isolation have only modest interrater reliability. Additionally, our ground truth annotations were derived from human annotations. Therefore, they were not independent, and our method for estimating human performance (by comparing each individual annotator to the final ground truth) was biased in favor of overestimating human performance. Still, it was important to have a baseline against which to measure the performance of our models, and makes it even more impressive that the models (which were independent of the ground truth annotations) performed comparably to human annotators.

Our experiments also showed that the language models finetuned under our pipeline can achieve near-human and human-like performance on clinically meaningful epilepsy-related tasks. We found the best performing combination of models to be Bio_ClinicalBERT_FT_ and RoBERTa_FT_ for text classification and text extraction, respectively. We observed that model performance on the classification question was slightly worse than human performance (although the model was still quite accurate), and model performance on the text extraction questions was equivalent to human performance. We hypothesize that this discrepancy may be in part because of the more complex logical reasoning required for the classification task. For example, if a note says “the last seizure occurred in June 2015,” it requires no inference to extract this text as the answer to Question 3, “When was the patient’s most recent seizure?” Meanwhile, correctly classifying the patient as seizure-free (Question 1, “Has the patient had recent seizures?” = no) requires knowledge of when the note was written, whether that date is more than 1 year after June 2015, and that 1 year is the criteria for a recent seizure. Previous work has shown that logical reasoning and temporal reasoning remain challenging for language models.^22–27^

Such high performances on clinical datasets have been seen in other studies, though on different datasets and tasks. For example, Alsentzer et al used Transformer Models to achieve 82.7% accuracy on the MedNLI dataset,^28^ which measures how well a model can infer relationships between a hypothesis and premise using clinical texts.^12^ Additionally, both Alsentzer et al^12^ and Yang et al^29^ used Transformers to achieve F_1_ scores between 75 and 95 on various i2b2 and/or n2c2 clinical challenges. Previous work has also been done to apply NLP to epilepsy clinical notes: Fonferko et al^30^ used a rules-based NLP method to extract seizure frequency, among other information, from 200 clinical letters, achieving an F_1_ score of 66% on a per-item basis and 72% on a per-letter (ie, per-note) basis. Our methods differed from theirs in several aspects, especially in the manual steps required. In addition to preparing their data, they used manually coded rules for detecting statements of seizure frequency and large dictionaries of search terms to handle the intricacies of language and the various ways that seizure frequency can be represented in the text. In contrast, our manual efforts were confined solely to create and annotate our data. The language model then uses that data to learn and understand the language in a more generalizable fashion. It is also important to note that the F_1_ scores reported in our and other studies may measure performance differently, depending on the NLP task and method, limiting direct comparisons between studies.

The findings of our ablation studies contrast with some of the literature. Han and Eisenstein,^17^ which inspired our pipeline, combined unsupervised domain adaptation with supervised task adaptation to improve BERT’s performance on sequence tagging in difficult domains. They devised 3 BERT models—a frozen BERT baseline, a task-tuned BERT, and a BERT with both domain tuning and task tuning. They found that sequentially adding these finetuning steps improved model performance over the baseline. Meanwhile, Soni and Roberts^18^ found that using SQuAD as an intermediate dataset improved clinical question answering performance for several models. In contrast, we found that only domain tuning and the annotated training set proved to be helpful, and only the annotations proved to be critical to model performance. Task tuning using intermediate datasets had no significant impact on the final performance of our models.

Our study does have some limitations. Our clinical notes are sourced from a single healthcare center and as such, the generalizability of our results to other centers is unknown and should be tested in future research. Similarly, we annotated, trained, and tested on specific neurology notes; our model may experience decreased performance when applied to notes from other departments. However, these problems can be solved with additional finetuning on a small dataset sourced from either the new institution or department, in the manner that we have described within this article. Additionally, our choice in models was constrained by the limitations of our 16 GB Nvidia P100 GPU. This prevented us from using different, larger models, such as Longformer,^31^ which has been found to outperform RoBERTa on long document tasks. This problem can be resolved by using a more powerful GPU in the future.

Our definition of seizure freedom (since last office visit or within 1 year) was also pragmatic, and reflects clinical practice for measuring this outcome measure, but from a machine learning perspective was arbitrary and may have affected the accuracy of our classification models. We also captured and lumped together the frequency of all types of seizures. For clinical purposes, some types of seizures are more severe (eg, bilateral tonic-clonic convulsions) and may require different clinical management than milder types (eg, focal aware “auras,” or myoclonic jerks). For patients with multiple types of seizures, extracting different frequency information for different seizure types should be addressed in future research. Additionally, copy-and-pasted information, a challenge for current electronic medical record systems, sometimes led to internally contradictory text within a single note. These are inherent challenges in NLP of free-text medical notes and will remain a challenge until medical records systems further constrain data entry or capture clinical data in more discrete, minable forms instead of prose text.

In the future, we hope to use our model to extract seizure freedom and seizure frequency data from clinical notes in real-time practice to improve clinical decision-making. We also plan to perform large-scale retrospective epilepsy research using data from UPHS and potentially from collaborating centers with the push of a button.

## CONCLUSION

In conclusion, our novel NLP pipeline allowed models to extract clinically important outcome measures, seizure freedom, and seizure frequency for patients with epilepsy, with accuracies comparable to that of human annotators. This tool will enable investigators to mine outcome data from clinical note text at scale to facilitate clinical research and decision-making. The amount of training data required to fine-tune the model was smaller than expected, suggesting that this tool can be adapted to other applications in the future with modest preparation.

## FUNDING

This research was funded by the National Institute of Neurological Disorders and Stroke 1DP1 OD029758; by the Mirowski Family Foundation; and by contributions from Jonathan and Bonnie Rothberg. CAE is supported by the National Institute of Neurological Disorders and Stroke of the National Institutes of Health Award Number K23NS121520; by the American Academy of Neurology Susan S. Spencer Clinical Research Training Scholarship; and by the Mirowski Family Foundation. DR’s work was partially funded by the Office of Naval Research Contract N00014-19-1-2620.

## AUTHOR CONTRIBUTIONS

KX, RSG, SNB, BL, CAE, and DR conceived, designed, and/or implemented the study; KX, RSG, SNB, MW, RM, JK, BL, CAE, and DR implemented initial investigations and experiments; KX, RSG, ECC, and CAE. developed the annotation protocol of the study and performed pilot annotation studies; KX, ECC, COG, JMB, PDG, NJG, ASG, TJ, AK, CVK, JMP, ARP, BHS, DW, and CAE performed annotations; KX and CAE adjudicated annotations; KX, COG, and CAE analyzed and interpreted the data from the study; KX, RSG, BL, CAE, and DR drafted and edited the article.

## SUPPLEMENTARY MATERIAL

Supplementary material is available at *Journal of the American Medical Informatics Association* online.

## CONFLICT OF INTEREST STATEMENT

None declared.

## DATA AVAILABILITY

The clinical data in this study cannot be publicly shared due to patient privacy concerns. However, our code is available on Github at https://github.com/penn-cnt/Extracting-Clinical-Information-From-Epilepsy-Clinic-Notes. We also provide a Zenodo DOI for our code at: https://doi.org/10.5281/zenodo.5838235. Our models can be accessed through the Huggingface Hub under the names: CNT-UPenn/Bio_ClinicalBERT_for_seizureFreedom_classification and CNT-UPenn/RoBERTa_for_seizureFrequency_QA.

## Supplementary Material

ocac018_supplementary_data
