# Supplementary material for: Extracting seizure frequency from epilepsy clinic notes: a machine reading approach to natural language processing
Source: J Am Med Inform Assoc. 2022 Feb 22;29(5):873–81. doi: 10.1093/jamia/ocac018 (PMC9006692; doi:10.1093/jamia/ocac018)
Supplement: ocac018_supplementary_data [file ocac018_supplementary_data.zip › Official_Inception_Annotation_Protocol_Jun182021.pdf]

Official Annotation Protocol

I. Definitions:

A. Epileptic event: Any event that may be related to epilepsy or seizures including, but not limited to the various types of seizures; auras; myoclonus; and staring spells.
   - This includes known or suspected non-epileptic events.

B. **Recent** epileptic event: An event that occurred since their last visit or within the past year, whichever is closer to the note's date.
   - For example: "The patient last visited us three months ago. The patient had a seizure nine months ago but had no seizures since their last visit." In this scenario, there are no recent seizures, since the patient did not have a seizure since their last visit, which is closer to the present time.
   - The date the note was written can be used to infer the answer. For example, if the note is dated 08/01/2015 and the patient's last seizure was on 01/01/2015, this **does** meet the definition of a recent epileptic event (assuming no additional information about seizures since last visit).

C. Last Occurrence: The last occurrence of an epileptic event is the most recent occurrence of that event. It does not mean that the patient is cured of that type of event.
   - For example: "They had a convulsion since their last visit, on January 23rd" would be a last occurrence.

D. Seizure Calendar: A list of the number of events that occur in each month over a span of several months.
   - For example: "Jan: 2, Feb: 3, Mar: 1, Apr: 0" is a seizure calendar.
   - Seizure calendars are at the month "level". A list of dates that events occurred on is **NOT** a seizure calendar (i.e. "Seizures occurred on 9/12, 9/14, 9/16" would not be a calendar).

II. Annotation

At the beginning of an annotation session, perform step 1:

1. Set annotation settings (Perform this step only once at the start of each annotation session)
   a. Inside any annotation document, click the settings icon on the toolbar (right side)
   b. Under the General Display Preferences, set the following:
      i. Editor: brat (line-oriented).
      ii. Page size: 50 rows.
   c. Press Save.

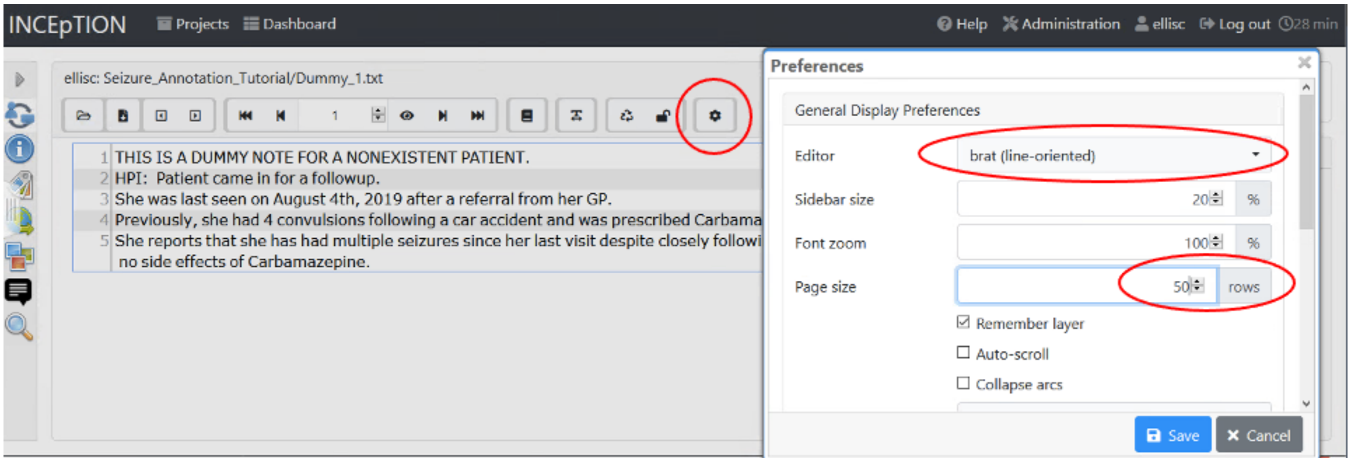

Within each document that you annotate, perform the following steps:

2. <u>Annotate the "HasSzs" Layer</u>: Indicate the presence or absence of recent epileptic events.
    a. **Select "HasSzs"** from the 'Layer' dropdown menu.
        - If the patient clearly is or is not having recent epileptic events:
            1. Click and drag to **highlight the first minimum necessary span of text** that most strongly indicates this.
            2. **Select either "Yes" or "No"** from the "HasSeizures" feature selection window on the right.
        - If it is unclear or undefined whether the patient is having recent epileptic events.
            1. **Highlight the first word of the document** and **label it with the "Unspecified"** category from the feature selection window on the right.

3. <u>Annotate the frequency of epileptic events.</u>
    - We consider only the following two classes of frequencies.
        - Class 1 - *Positive Quantitative Frequency*: A statement that is indicative of a nonzero pattern of occurrence or an ongoing set of occurrences (extends to the present). Examples:
            1. "He has seizures **twice per month."** (This indicates a pattern)
            2. "She had **six seizures in the last month."** (This indicates occurrences that extends to the present)
            3. "They had **three staring spells since the last visit."** (This indicates occurrences that extends to the present)
            4. **Any seizure calendar** - "Jan: 2, Feb: 3, Mar: 1, Apr: 0" (These expand on a pattern of occurrences and often extend up to the present date)
            5. On the other hand, an example like "They had nine seizures in September" is **NOT** a Positive Quantitative Frequency because it does not imply a pattern, and does not extend to the present.
        - Class 2 - *Explicit Last Occurrence*: Any statement of zero frequency or of a last occurrence from which an explicit date can be assigned to. Examples:
            1. Patient had **no seizures since January.**
            2. Patient's **last seizure was in January.**
            3. Her **last convulsion was two week ago.**
            4. His **last seizure was in 2015.**
            5. She had a **seizure in September, and no seizures since**.

Additional rules about annotating frequency:

- All seizure calendars should be annotated as a Positive Quantitative Frequency. Some calendars may have Explicit Last Occurrence information, but please ignore that and annotate the calendar as one PQF.
- For a given type of epileptic event, we want either the most recent or current of these classes. We are not interested in historical data, such as frequency information for seizures that are no longer relevant to the current status of the patient.
    - For example: "Patient is having **seizures once a month**. In the past, they were having seizures every day." In this case, we only want to annotate the first sentence, not the second sentence which does not contain the most recent/current information.
- We are not interested in single dates of seizures or lists of dates for seizures if they do not pertain to a frequency or a last occurrence.

- For example: "**Their last seizure was last week**. Prior to this, there was a seizure two months ago and another seizure on June 15." In this case, only annotate the most recent seizure as an Explicit Last Occurrence.

- Some events may be specified by both a frequency and an explicit last occurrence, in which case we annotate both statements.
  - For example: "Patient has **three seizures per month**. Their **last seizure was in January.**" In this case, you would annotate both a Positive Quantitative Frequency, and an Explicit Last Occurrence.
- Frequency annotations often overlap with HasSzs annotations.

How to annotate:

a. **Select "SeizureFreq"** from the 'Layer' dropdown menu.
b. Click and drag to **highlight the first minimum necessary span of text** that captures both the number/rate of events and the time frame (e.g., "5 seizures in the past three months").
   - You do not need to include the description of the event that this frequency refers to.
     - For example: "That patient has tonic-clonic seizures once a month" – you should only select the text "once a month".
b. **Select the corresponding class of "Seizure Frequency"** from the feature selection window on the right.
c. Repeat steps for each (recent) frequency class associated with an epileptic event.
d. If there are multiple epileptic events with their own frequencies, repeat these steps for each such frequency.

4. If there is more than one type of epileptic event with an associated frequency, or if an epileptic event has more than one associated frequency class, then proceed to step 5. Otherwise, skip to step 6.

5. <u>Annotate types of epileptic events.</u>
   a. For each epileptic event with an associated frequency:
      i. **Select "SeizureType"** from the 'Layer' dropdown menu.
      ii. **Highlight the span most proximal to the respective frequency** if applicable, that identifies which type of epileptic event is being described.
      iii. **Identify the most relevant type of epileptic event** in the "Type of Seizure" feature selection window on the right.
   b. Connect each type of event to its associated frequency or frequencies:
      i. **Select "TypeToFreq"** from the 'Layer' Dropdown Menu.
      ii. Highlight the span(s) of text corresponding to these frequencies again.
      iii. Highlight the span(s) of text corresponding to the associated epileptic events again.
      iv. **Click on a "TypeToFreq" label and drag** to the other to connect the two.
      v. If an event has more than one associated frequency class, **connect all frequency classes to the event** in the same manner.

6. <u>Finalize the annotation</u>: When you are finished with a document, **press the lock button** to finish annotating the current document.

III. FAQs

1. If a patient has not had an epileptic event since their last visit (e.g., “No convulsions since last visit”), is this an Explicit Last Occurrence frequency?
    a. No. We only know that the patient has not had an event since their last visit. We do not know the date on which their event last occurred. In this case, you should annotate the HasSz layer, and do not annotate anything for the SeizureFreq layer.
2. How do you annotate a sentence that invokes multiple types of seizures for a single frequency? For example, “Patient has staring spells, myoclonus and auras twice per month”.
    a. Annotate the frequency normally (positive quantitative in this example).
    b. Annotate each type of seizure.
    c. Use TypeToFreq to connect each type of seizure to the frequency (this will create a long chain).
3. If a sentence is indicative of the HasSzs layer, and contains a frequency, should I annotate both layers and have an overlapping annotation?
    a. Yes, there will be times your annotations will overlap or intersect other annotations. In fact, many times the text that you annotate for the HasSzs layer will also need to be annotated for the SeizureFreq layer.
4. How should I annotate “They had 3 seizures since the last visit. Five years ago, they had 5 seizures per month.”?
    a. For a given frequency class, we want only the most recent or relevant span of text. You would thus only annotate “3 seizures since the last visit” as a Positive Quantitative Frequency.
5. Do I need the TypeToFreq layer if there is only one frequency with an associated epileptic event mentioned?
    a. No. Only annotate type-to-frequency if there is more than one epileptic event with a frequency.
6. If there is no info on whether they are currently seizing, what should I annotate ?
    a. Select the first word of the document and annotate it with HasSz = Unspecified.
7. If there is no frequency info, should I just leave the frequency layer blank?
    a. Yes.
8. If a note describes "occasional seizures" or “has ongoing seizures” without additional info, is this a frequency?
    a. No. It does not fit into any of the two classes of seizure frequencies that we are considering.
9. What should we do with non-specific events that could possibly be seizure (e.g., "periods of lost time")? What about uncertain events (e.g., Patient may have had a GTC during the night)
    a. According to the above definition of “epileptic event”, such non-specific events that are related to epilepsy should be annotated, and such uncertain events should be annotated as if they were definitively real.
10. When should I label an epileptic event as having “unspecified” type?
    a. Whenever the *entire* passage does not contain sufficient information to determine the type of epileptic event.
    b. It is possible that context from previous or later sentences can be used to identify the type of epileptic event, in which case the event type should be specified accordingly. However, in this scenario, you should still highlight the span, most proximal to the respective frequency, mentioning the specific epileptic event.
11. Should we count “no seizures recently” as frequency information?
    a. No. This is not a Positive Quantitative Frequency or an Explicit Last Occurrence.
12. Should we count “last seizure 2014” as frequency information?
    a. Yes. This is an Explicit Last Occurrence.
13. Should annotations span multiple sentences?
    a. Generally, no, you should be highlighting only the minimum span of text necessary for the annotation and try to keep annotations within a single sentence.
14. How should I annotate “Patient had 4 seizures since the last visit. Three were GTC, while one was FAS.”?

   a. Annotate “4 seizures since the last visit”, “Three”, and “one” each as Positive Quantitative Frequency.
   b. Annotate “seizures”, “GTC”, and “FAS” as “Unspecified”, “Convulsions...”, and “FAS, SPS”, respectively.
   c. Annotate each aforementioned span with a TypeToFreq Layer.
   d. Using the TypeToFrequency layers, connect “seizures” to “4 seizures since the last visit”; “GTC” to “Three”; and “FAS” to “one”.

15. If multiple epileptic event types are described, but only one with a corresponding frequency, do we need TypeToFreq?
    a. No. You only need TypeToFreq if there is more than one epileptic event that has an annotated frequency.
16. How should I annotate “No convulsions since January. The patient continues to have auras once per week and myoclonic jerks three times a month.”?
    a. Annotate HasSzs = Yes because the patient is having epileptic phenomena (auras and myoclonic jerks).
    b. Annotate the three different frequencies stated in the text.
        i. “once per week” as Positive Quantitative Frequency
        ii. “three times a month” as Positive Quantitative Frequency
        iii. “No convulsions since January” as Explicit Last Occurrence.
    c. Annotate the three different seizure types that the patient is having.
        i. “auras” as FAS (focal aware seizures)
        ii. “myoclonic jerks” as myoclonic
        iii. “convulsions” as Convulsions, Grand Mal, BTC, GTC, Tonic-Clonic
    d. Because there are multiple different seizure types and multiple frequencies, use the “TypeToFreq” layer to connect the aforementioned spans to their respective types and frequencies.
