# Supplementary material for: Extracting seizure frequency from epilepsy clinic notes: a machine reading approach to natural language processing
Source: J Am Med Inform Assoc. 2022 Feb 22;29(5):873–81. doi: 10.1093/jamia/ocac018 (PMC9006692; doi:10.1093/jamia/ocac018)
Supplement: ocac018_supplementary_data [file ocac018_supplementary_data.zip › Revised Supplement Clean Copy.docx]

**SUPPLEMENTAL MATERIALS**

**Extracting Seizure Frequency From Epilepsy Clinic Notes: A Machine Reading Approach To Natural Language Processing**

Kevin Xie, Ryan S. Gallagher, Erin C. Conrad, Chadric O. Garrick, Steven N. Baldassano, John M. Bernabei, Peter D. Galer, Nina J. Ghosn, Adam S. Greenblatt, Tara Jennings, Alana Kornspun, Catherine V. Kulick-Soper, Jal M. Panchal, Akash R. Pattnaik, Brittany H. Scheid, Danmeng Wei, Micah Weitzman, Ramya Muthukrishnan, Joongwon Kim, Brian Litt, Colin A. Ellis*, Dan Roth*

*Contributed equally

**SUPPLEMENTAL METHODS**

**selecting and pre-processing progress notes**

We selected progress notes authored by epilepsy specialists within the Neurology Department at UPHS to improve the probability that the progress notes related to epileptic events. We excluded attending attestations to trainee notes, as they contain an abbreviated amount of information. We extracted only text under the History of Present Illness (HPI) and Interval History sections as independent “paragraphs,” sections verified by expert clinicians to be the most likely to contain the most recent and relevant seizure-related information.

We truncated these paragraphs to 1506 characters under the assumption that a token would represent three characters on average, and that the header of each paragraph (either History of Present Illness, HPI, Interval History, or similar) would be at most 30 characters. We then appended the statement “This note was written on [date written],” where [date written] indicates the date the note was written, to the front of each paragraph. A finalized paragraph would thus be of the form “This note was written on [date written]. [Header]: [Note text truncated to 1506 characters],” and would be tokenized as such.

We measured the distribution of the tokenized lengths of the untruncated paragraphs, and the truncated, finalized paragraphs to demonstrate the necessity and effectiveness of our paragraph preparation process. We used the BERT tokenizer to perform the tokenization. It is evident that many untruncated documents exceeded the 512 token cap; however, once truncated, only five paragraphs exceeded 512 tokens, even with the appended sentence containing the date the note was written (Supplementary Figure 1).

**annotation specifics**

Several pilot annotation studies were launched on the INCEpTION annotation platform to create an annotation protocol and to refine the text extraction and pre-processing methodology. In the final annotation protocol (see supplementary material – Official Annotation Protocol), annotators were instructed to use the INCEpTION platform to annotate the first span of text that suggested a patient was event-free or having recent events and mark it as such. We defined events to include the following common epileptic phenomena: Jerks, Auras, Seizures, Staring, Events, Episodes and Spells. Annotators then identified all spans of text that denoted a patient’s event frequency or the approximate date of their most recent event(s). If a patient was found to have more than one type of event, Annotators were to differentiate between each type by annotating the type of event and connecting it to its associated frequency or date of last occurrence. Finally, to ensure that we were annotating clinically recent and relevant information, we instructed annotators to ignore text that was obviously copy-forwarded (text that a clinician has copied over from the previous note).

Ultimately, 15 initial annotators were recruited for the task, including clinical epilepsy nurse practitioners, neurology residents, epilepsy fellows, and attending epileptologists, and non-clinician graduate students or personnel working in a lab focused on Epilepsy-related research. The annotators were uniformly split into five groups of three such that each group had at least one clinician and at least one non-clinician annotator. Each annotation group received 200 of the 1000 extracted paragraphs, such that no paragraph would be annotated by more than one group. Each annotator was trained on the annotation protocol and instructed to annotate all 200 paragraphs so that each paragraph would have three unique annotations for final adjudication. An artificial checkpoint was introduced after each annotator finished 25 of their 200 paragraphs to ensure consistent and high-quality annotations; if needed, annotators were given feedback or additional training during this checkpoint.

One annotator was unable to begin annotations, while another was unable to finish annotations. The former’s annotations were done by an annotator from a different group, while the latter’s annotations were completed by a newly recruited annotator. The final annotator composition consisted of seven clinicians (epilepsy nurse practitioners, neurology residents, epilepsy fellows, and attending physicians) and eight non-clinicians (graduate students or personnel working in a lab focused on epilepsy-related research).

**SUPPLEMENTAL RESULTS**

We observed that the models performed differently on the text extraction task depending on whether notes did or did not contain a ground truth answer to the question (Supplementary Figure 2). When no answer existed, performance was excellent for all models (all median F1 values > 0.90), as well as for human annotators. In contrast, for notes that contained an answer to the question, model performance was relatively lower (median F1 values 0.695 – 0.733), but exceeded that of most human annotators, who were highly variable in performance. For the classification question, too few notes had no answer (7.8%) to allow for meaningful subanalysis.

We also examined model performance in our ablation studies, stratified by whether an answer existed in each note. Although performance was lower overall in notes where an answer existed than in notes where an answer did not exist, the pattern of ablation results was similar in both categories of notes, with annotations being the only critical fine-tuning step (Supplementary Figure 3). Using a two-sided Mann-Whitney U Test, we found that only the annotations were statistically significant in affecting model performance when separately considering the case where the answer exists, and the case where it did not (p-values 0.011 and 0.008, respectively).

Performance on the text extraction task plateaued earliest for notes where no answer existed (Supplementary Figure 4). In contrast, for notes where an answer existed, this plateau was achieved more gradually, indicating that additional annotations provided incremental benefit for this subset of notes. However, even in this subset, 91.2% of the maximum performance was achieved with 20% of the training set (140 notes).

*Supplemental Figure 1: Distribution of token lengths using the BERT tokenizer before and after truncation and processing. Truncated paragraphs consistently stayed under the 512 token limit, while many untruncated tokens exceeded the limit. The vertical red dashed line denotes the 512 token limit, while the orange and blue histograms denote the truncated and untruncated paragraphs, respectively.*

*Supplemental Figure 2: Human and machine performance for extracting text from clinical notes (Q2 and Q3 combined), stratified by whether a ground truth answer existed. F_1_ scores were higher for when no answer existed than when an answer existed, for both human annotators and all models. Gray points denote individual annotator performance, while green, orange, and purple points denote individual Bio_ClinicalBERT_FT_, RoBERTa_FT_, and BERT_FT_ seeds, respectively. Box plots show median and quartile ranges of values.*

*Supplemental Figure 3: Performance of ablated models for extracting text from clinical notes, stratified by whether a ground truth answer existed. We conducted two-sided Mann-Whitney U Rank Tests and found that annotations are essential to model performance (p-values of 0.008, 0.011, and 0.008). MLM is only significant overall (p-value 0.032), but not to each scenario individually. Green, orange, purple and gray points denote the full model, the model without MLM finetuning, the model without SQuADv2, and the model without annotations, respectively. Box plots show median and quartile ranges of values.*

*Supplemental Figure 4: Influence of training set size on model’s ability to extract text, stratified by whether an answer exists. Training set size was most important on improving the model’s ability to extract existing answers, but overall, only 10% of the training data was required to achieve strong results.*
