# Supplementary figures and images for: Extracting seizure frequency from epilepsy clinic notes: a machine reading approach to natural language processing
Source: J Am Med Inform Assoc. 2022 Feb 22;29(5):873–81. doi: 10.1093/jamia/ocac018 (PMC9006692; doi:10.1093/jamia/ocac018)

Distribution of Tokens per Paragraph

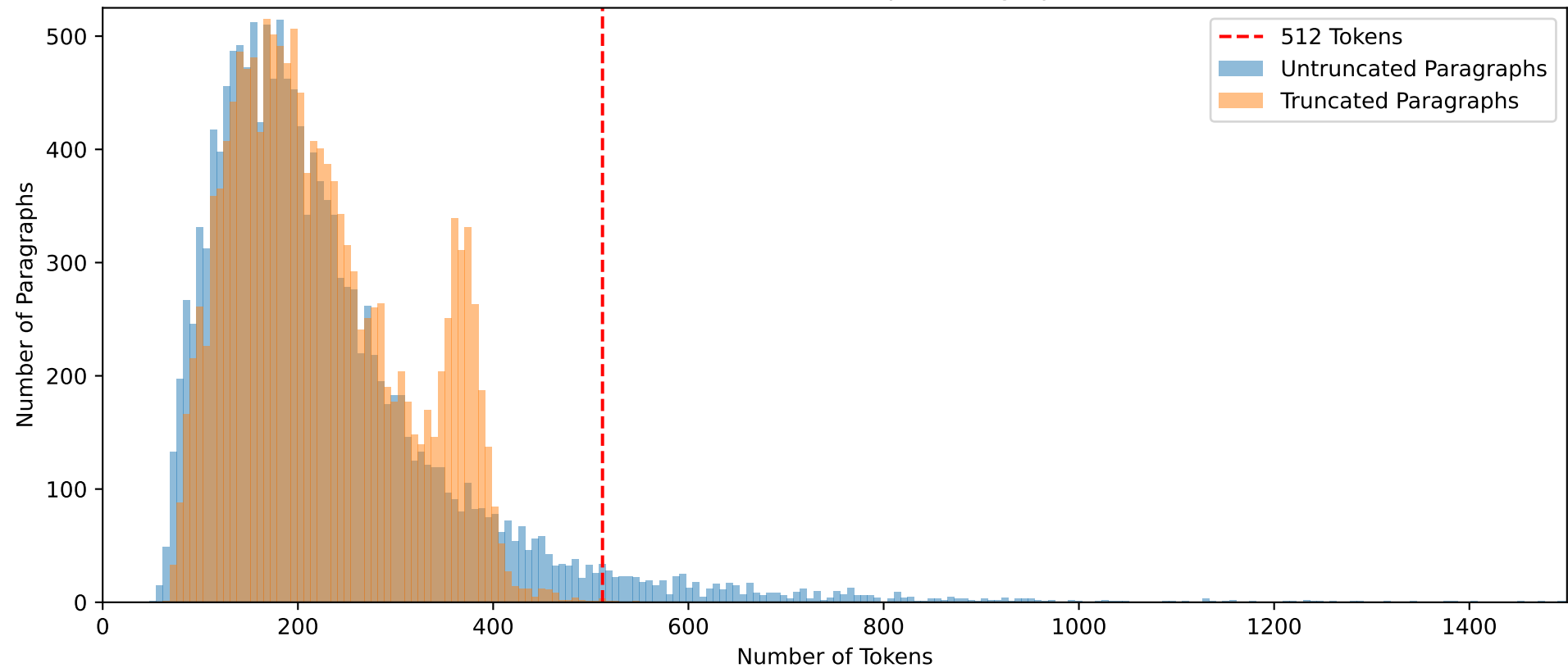

Supplement: ocac018_supplementary_data [file ocac018_supplementary_data.zip › Supplemental_Figure_1.pdf]

Human vs. Machine Performance on Extracting  
Seizure Frequencies and Occurrences

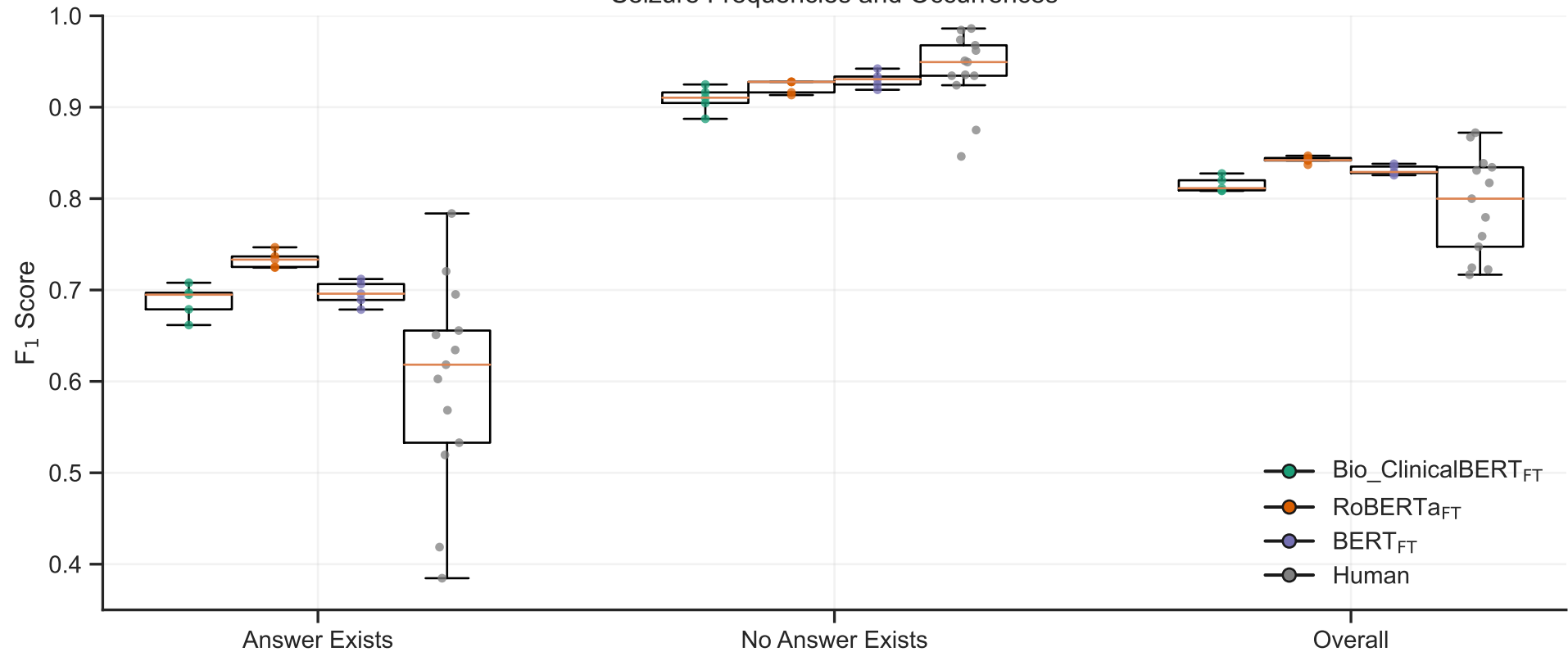

Supplement: ocac018_supplementary_data [file ocac018_supplementary_data.zip › Supplemental_Figure_2.pdf]

F<sub>1</sub> Score for Extracting Seizure Frequencies  
and Occurrences Following Ablation

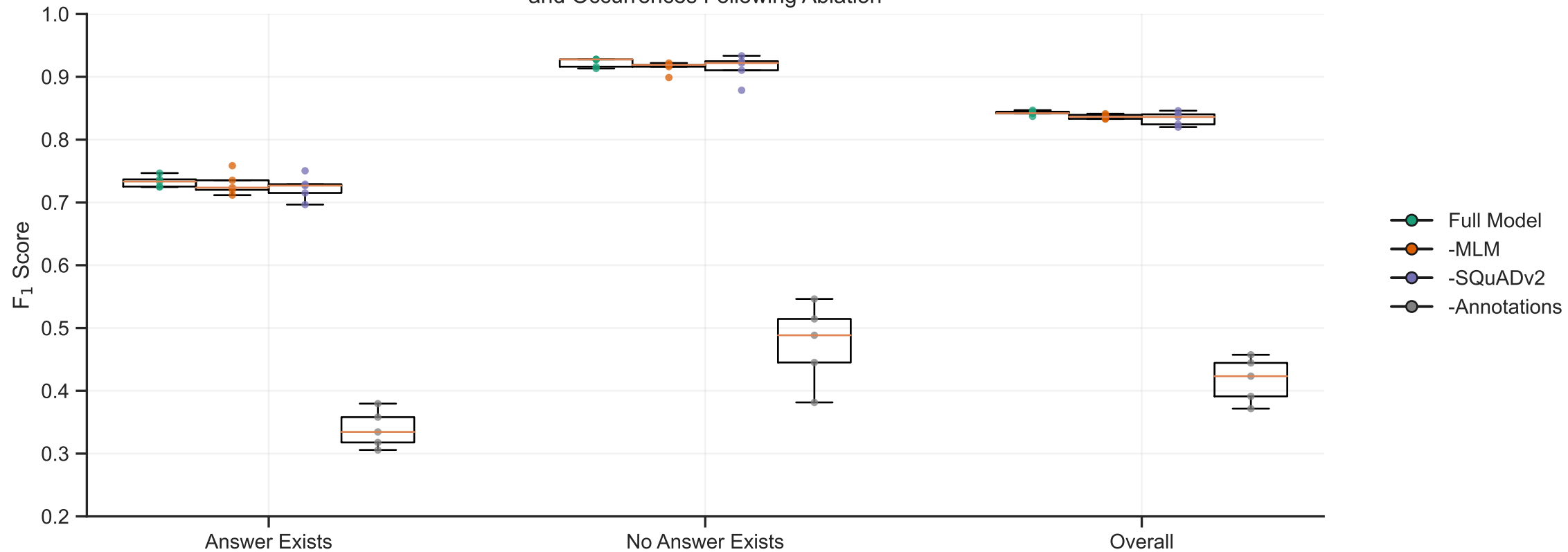

Supplement: ocac018_supplementary_data [file ocac018_supplementary_data.zip › Supplemental_Figure_3.pdf]

F<sub>1</sub> Score for Extracting Seizure Frequencies  
and Occurrences vs. Training Set Size (95% CI)

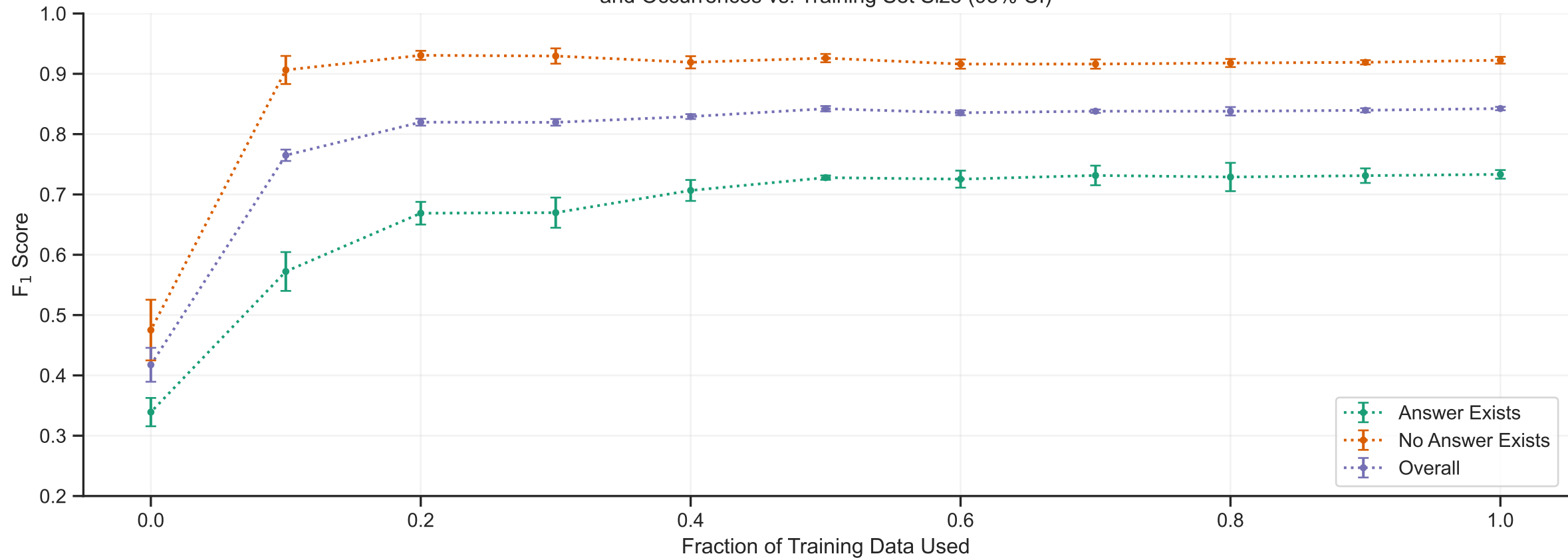

Supplement: ocac018_supplementary_data [file ocac018_supplementary_data.zip › Supplemental_Figure_4.pdf]
